# Combined clinical and radiological remission of rectovaginal fistulas using fractional CO2 vaginal laser: a case series and medium-term follow-up

**DOI:** 10.1186/s13104-023-06666-8

**Published:** 2023-12-19

**Authors:** Denise Gasparetti Drumond, Camila de Moraes Sarmento Condé, Júlio Maria da Fonseca Chebli, Liliana Andrade Chebli, Samuel Drumond Esperança, Neila Maria de Góis Speck

**Affiliations:** 1 Department of Surgery, Faculty of Medicine, Universidade Federal de Juiz de Fora, Rua Doutor Waldyr Lorentz, 11, Juiz de Fora, MG CEP: 36.037-752 Brazil; 2 Universidade Federal de Juiz de Fora, Juiz de Fora, Brazil; 3 Department of Medicine, Faculty of Medicine, Inflammatory Bowel Disease Center, Universitary Hospital, Universidade Federal de Juiz de Fora, Juiz de Fora, Brazil; 4 Hospital Israelita Albert Einstein, São Paulo, Brazil; 5 Department of Gynecology, Universidade Federal de São Paulo, São Paulo, Brazil

**Keywords:** CO2 laser, Treatment, Rectovaginal fistula

## Abstract

**Introduction:**

Despite the advances in surgical and clinical approaches, there is no consensus regarding the best line of treatment from rectovaginal fistula (RVF). Faced with a challenging scenario in the approach of RVF, the fractional CO2 laser receives attention as a possible form of treatment.

**Objectives:**

A single-center, prospective, open-label study evaluating the effectiveness and safety of laser therapy for RVF treatment.

**Subjects and methods:**

The total of 15 patients was recruited at the Juiz de Fora University Hospital between August 2018 and July 2022. Inclusion criteria were presence of clinically suspects RVF of any etiology confirmed by pelvic magnetic resonance image (MRI) and gynecological examination. Five fractional CO2 laser sessions with monthly interval followed by complete evaluation through clinical examination and pelvic MRI were performed for all patients after the completion of treatment. Analysis of sexual function before and after the treatment was performed using Female Sexual Quotient (FSQ).

**Results:**

The evaluation through physical examination showed no persistent inflammatory signs in the vagina for all patients. Additionally, 10 of out 15 (67.7%) patients achieved clinical remission of RVF symptoms, while 33.3% patients reported significant improvement. Of note, five patients who did not have previous sexual activity returned to regular sexual activity while seven patients who have baseline sexual activity had improvement in their sexual function as assessed by the FSQ. Three out of four ostomized patients had their ostomy reversed and remained without complains. All six patients with RVF secondary to Crohn’s disease reported a marked improvement in symptoms and sexual function. In seven (47%) patients radiological remission was confirmed by pelvic MRI.

**Conclusion:**

CO2 fractional laser can be considered a promising and safe therapeutic alternative for the management of RVF.

## Introduction

Rectovaginal fistula (RVF) is an abnormal tract that connects the lower gastrointestinal tract with the vagina. They can be divided into rectovaginal and anovaginal according to location, with rectovaginal affecting the upper two-thirds of the vagina and the rectum and anovaginal affecting the lower third of the vagina and the anal canal. Despite this classification, many authors use the term “rectovaginal” for both [1, 2]. It most frequently results from obstetric trauma, radiation damage, Crohn’s disease (CD) [3, 4], difficult hysterectomies, extension or rupture of perirectal, perianal and Bartholin's abscesses, and from any surgical procedures involving the posterior vaginal wall, anus, perineum or rectum [5]. Women suffering from RVF present mainly with uncontrollable passage of gas or feces from the vagina. A malodorous vaginal discharge and fecal soiling of the undergarments are also common complaints. Occasionally, a small fistula may be asymptomatic [4]. Diagnosis is made on vaginal, rectal and anal sphincter examination. The use of vaginal and anorectal speculum, colposcope, enema, endoanal ultrasound, and pelvic magnetic resonance imaging (MRI) may aid on diagnosis [4, 6].

For women with small fistulas and minimal symptoms, nonsurgical management is appropriate. Otherwise, surgical approaches to RVF repair are dictated by fistula etiology. Fistulas should undergo local repair via transvaginal, transanal, transsphincteric, or transverse transperineal approach. Also, a transabdominal approach is available. Patients with RVF due to CD should not undergo repair until adequate medical control of their disease has been achieved. Thus, there are numerous therapeutic approaches, but unfortunately with limited responses [7–9]^.^

Faced with a challenging scenario in the approach of RVF, the fractional CO2 laser should be considered as a possible form of adjuvant or primary treatment. Studies by Zerbinati and colleagues demonstrated the action of CO2 laser on vaginal tissue, promoting tissue remodeling with increased mucosal thickness through action with heat shock protein (HSP) and cytokines that promote fibrogenesis through the activation of fibroblasts and consequent production extracellular matrix; stimulating both the angiogenesis, with the proliferation and migration of endothelial cells; and re-epithelialization through epidermal growth factor activation [10, 11].

In a pilot study in 2019, Drumond and colleagues found complete occlusion of a rectovaginal fistulous path in a woman with CD, after performing 3 sessions of intravaginal fractional CO2 laser. In that report, the use of the biological therapy (i.e., Infliximab) was maintained as a drug for continuous treatment of the CD [12]. The same authors, in 2021, following five women with RVF who underwent 5 sessions of fractional CO2 laser, achieved complete closure of the fistulous tract in three and partial closure in two women. Two women, previously ostomized, had their ostomy reversed. Of these, four patients resumed sexual function as evaluated by female sex quotient [13, 14].

Within this context, the present study evaluates the rate of combined clinical and radiological remission of RVF induced by therapy with fractional CO2 vaginal laser, as well as the sexual function as evaluated by FSQ of women before and after treatment and therapy safety.

## Methods

### Study design

We conducted a single-center, prospective, open-label study on patients with RVF undergoing the vaginal laser therapy. The present study and cases reports were approved by the institutional review board and ethics committee of Juiz de Fora University Hospital, whose number of National Commission for Research Ethics (Brazil) 5.636.932.

### Study population

Patients were recruited at the Juiz de Fora University Hospital between August 2018 and July 2022. Inclusion criterion was presence of clinically suspect RVF of any etiology confirmed by pelvic MRI and gynecological examination. Exclusion criteria were age younger than 18 years or older than 70 years, active genital malignancy, severe genital infection, breastfeeding women, and pregnancy. All patients supplied written informed consent before study inclusion.

### Study interventions

At entry, the eligibility criteria were assessed, and medical history was recorded, including age, gender, race, etiology, duration and clinical presentation of the RVF, presence or absence of active sexual activity, sexual function, previous surgery for RVP (yes/no) and baseline medications. In addition, a physical exam using a speculum and a Pelvic MRI with venous administration of a gadolinium-based contrast agent were performed at inclusion.

For evaluating the sexual function, we used the validated Brazilian version of the Female Sexual Quotient (FSQ) questionnaire [14]. FSQ is a brief, easy-to-apply questionnaire useful for screening female sexual dysfunction. This questionnaire includes 10 questions, each of which must be answered on a scale of 0–5. The sum of the 10 answers should be multiplied by 2, resulting in a total index ranging from 0 to 100. The seventh question requires a different treatment, i.e., the value of the answer given (from 0 to 5) must be subtracted from 5 to achieve the final score for this question. Higher values indicate better sexual performance/satisfaction, namely, 82–100 points: good to excellent; 62–80 points: regular to good; 42–60 points: unfavorable to regular; 22–40 points: bad to unfavorable; and 0–20 points: null to bad [14, 15].

Following confirmation of the RVF diagnosis patients were referred to undergo five vaginal laser sessions, fractionated at weeks 0, 4, 8, 12, and 16 using the CO2 laser (SmartXide2 V2 LR, Monalisa Touch; DEKA). The settings were: DOT (micro-ablative zone) power of 40W, dwell time of 1000 µsec, DOT spacing of 1000 µm, and a 4 Stack. The laser probe was gently inserted into the vagina, without using the speculum, lubricants or topical anesthetics. The treatment takes 4 min. The patient was instructed to avoid sexual activity within 3 days of the laser procedure if it were the case.

Patient’s follow-up included clinical visit with physical exam using a speculum on 4, 8, 12, 16 and 20 weeks after the first laser session. In addition, patients were instructed to report at once any adverse event following vaginal laser sessions. All gynecological evaluations before and after treatment of the RVF were performed by the same experienced gynecologist (DGD).

Between weeks 20 to 24 after ending the five sessions of vaginal laser, patients were asked about the presence or absence of active sexual activity, sexual function, and satisfaction with laser therapy (satisfied, very satisfied or dissatisfied). Furthermore, a pelvic MRI was schematized in order to assess remission or persistence of the fistulous path. The same experienced radiologist, who was not aware of clinical data and patient outcomes, examined all MRI images before and after treatment (Condé CMS).

### Study outcomes

The main outcomes measured were radiological and clinical remission of RVF, return of sexual activity, sexual function, patient’s satisfaction after treatment, and adverse events of therapy (therapy safety).

Clinical remission for RVF was defined as epithelization of the index external fistula opening (vaginal opening) compared to baseline associated with the absence of RVF-related symptoms. Radiological remission was defined by MRI non-identification of the fistulous track, with the presence of tissue with low signal intensity on T2-weighted images, inferring fibrosis and obliteration of the vaginal component of the fistula with the presence of tissue proliferation; radiological criteria for fistula improvement were reduction in the dimensions of the fistulous track and absence of enhancement by venous contrast, inferring improvement in the inflammatory process.

### Statistical analysis

Statistical analysis was performed using SPSS 20.0 (SPSS, Chicago, IL, USA). The quantitative variables are expressed as the median and range or as the mean ± standard deviation when normally distributed, and the categorical variables are expressed as absolute and relative frequencies. Comparison before and after the end of the fifth vaginal laser session on the same subjects that had baseline sexual activity was performed for FSQ scores using paired t test. For the purpose of comparison, the level of statistical significance was set to P < 0.05.

## Results

Fifteen female patients with diagnosis of RVF confirmed by clinical examination and pelvic MRI were consecutively treated with vaginal laser therapy and prospectively followed during a mean period of 27 months (range 7 to 48 months). The patients were between 29 and 69 years old (mean age 49), nine were white and six were no white. Four patients had rectovaginal fistulas and eleven had anovaginal fistulas. Crohn's disease was the most common cause of RVF (n = 6), followed by pelvic surgery (n = 4), perineal surgery (n = 2), unknown causes (n = 2) and obstetric trauma (n = 1). The mean duration of RVF before laser therapy was 30.1 months (range 6 to 120 months). The baseline symptoms included one or more of the following: vaginal burning, dyspareunia, passage of gas and feces from the vagina, foul-smelling vaginal discharge and tenderness to vaginal touch (Table 1).

**Table 1:** Demographics, clinical characteristics, and outcome after vaginal laser therapy in patients with rectovaginal fistulas

Variable	Age (years)	Race	Etiology	Duration of the fistula (months)	Baseline clinical manifestations	Baseline treatment	Baseline Sexual activity yes/no (FSQ score)	Sexual activity after laser therapy Yes/no (FSQ score)	Outcome	Pelvic MRI after laser therapy	Site of the fistula^a^/diameter before and after treatment
Patient 1	50	No white	Crohn’s disease	24	Vaginal burning, dyspareunia, foul-smelling discharge, significant vaginal tenderness to vaginal touch	Infliximab + prednisone 25 mg/day	Yes (40)	Yes (62)	Asymptomatic	Radiological improvement	Anovaginal 4 mm–2 mm
Patient 2	40	White	Pelvic surgery due to pelvic endometriosis	12	Foul-smelling minimal vaginal discharge, tenderness to vaginal touch, ostomized	None	No	Yes	Asymptomatic Ostomy reversal	Radiological remission	Rectovaginal 7,8 mm–0
Patient 3	29	No white	Pelvic surgery due to pelvic endometriosis	6 months	Foul-smelling minimal vaginal discharge, tenderness to vaginal touch, ostomized	None	No	Yes	Asymp-tomatic, ostomy reversal	Radiological remission	Rectovaginal 3 mm–0
Patient 4	38	No white	Vulvovaginal surgery due to bartholin	21	Vaginal burning, passage of gas and feces from the vagina, foul-smelling significant vaginal discharge tenderness to vaginal touch	Patient had been submitted to two previous fistulectomies	No	Yes	Occasional foul-smelling minimal vaginal discharge	Radiological improvement	Anovaginal 7.5 mm–2 mm
Patient 5	58	No white	Crohn’s disease	120	Vaginal burning, passage of gas and feces from the vagina, foul-smelling significant vaginal discharge tenderness to vaginal touch	Infliximab and azathioprine	No	Yes	Occasional foul-smelling minimal vaginal discharge	Radiological improvement	Anovaginal 3 mm – 1,7 mm
Patient 6	32	White	Unknown	24	Vaginal burning, dyspareunia discharge, foul-smelling significant vaginal, tenderness to vaginal touch	Patient had been submitted to three earlier fistulectomies	Yes (34)	Yes (76)	Asymptomatic	Radiological improvement	Rectovaginal 4 mm–0
Patient 7	36	White	Crohn’s disease	48	Vaginal burning dyspareunia passage of gas and feces from the vagina elimination of foul-smelling fluid vaginal touch was painful	Infliximab and azathioprine	Yes (20)	Yes (78)	Asymptomatic	Radiological remission	Anovaginal 4 mm–2 mm
Patient 8	31	White	Vaginal delivery with episiotomy	12	Vaginal burning dyspareunia passage of gas from the vagina elimination of foul-smelling fluid vaginal touch was painful	Previous fistulectomy	Yes (18)	Yes (90)	Passage of gas from the vagina elimination of foul-smelling fluid	Radiological improvement	Anovaginal 2.5 mm–2 mm
Patient 9	31	White	Vulvo-vaginal surgery due to bartholin	24	Dyspareunia passage of gas and feces from the vagina elimina-tion of foul-smelling fluid	Previous fistulectomy	Yes (74)	Yes (78)	Passage of gas from the vagina	Radiological remission	Anovaginal 3 mm–1.5 mm
Patient 10	47	White	Crohn’s disease	36	Elimina-tion of foul-smelling fluid Vaginal burning dyspareu-nia	Infliximab	Yes (32)	Yes (56)	Asympto-matic	Radiological remission	Anovaginal 3 mm–0
Patient 11	51	White	Unknown	48	Elimina-tion of foul-smelling fluid vaginal, touch was painful, dyspareu-nia passage of gas from the vagina	Antibiotic	Yes (30)	Yes (76)	Asympto-matic	Radiological remission	Anovaginal 3 mm–2 mm
Patient 12	69	White	Histerec tomy + annexec- tomy bilateral	9	Elimina-tion of foul- smelling fluid, ostomized	None	No	No	Asympto-matic	Radiological remission	Anovaginal 3.5 mm–0
Patient 13	68	No white	Rectosig- moidec- tomy due to rectal adeno-carcino-ma	8	Eliminati-on of foul- smelling fluid, ostomized	None	No	No	Asympto-matic	Radiological improvement	Anovaginal 4.4 mm–2.2 mm
Patient 14	39	No White	Crohn’s disease	12	Passage of gas from the vagina, elimina-tion of foul-smelling fluid	Infliximab and azathioprine	Yes (72)	Yes (72)	Passage of gas from the vagina	Radiological improve-ment	Anovaginal 3.3 mm–2.6 mm
Patient 15	39	White	Crohn’s disease	48	Elimina- tion of foul- smelling fluid vagina, touch painful, passage of gas from the vagina	Earlier fistulectomy, infliximab and azathioprine	No	Yes	Asympto-matic	Radiological improvement	Rectovaginal 3.7 mm–2.7 mm

*FSQ* female sexual quotient; *MRI* magnetic resonance imaging

^a^Rectovaginal fistula when it affects the upper two thirds of the vagina and rectum and Anovaginal fistula when it affects the lower third of the vagina and the anal canal

Seven patients had not sexual activity before laser therapy. Four patients had undergone previous fistulectomy surgeries and one of them had already undergone three unsuccessful fistulectomies (Table 1). All six patients with Crohn’s disease benefited in some way from the treatment. Four became asymptomatic and 2 had their complaints reduced. Closure of the fistula confirmed by imaging (RMP) was achieved in 2 cases and its dimensions were reduced in 4 cases. As for fistulas resulting from surgical interventions (6), 4 remained asymptomatic and 2 had an improvement in symptoms. Closure of the fistula confirmed by imaging (RMP) was achieved in 4 cases and its dimensions were reduced in 2 cases.

Four patients we could see passage of feces from the vagina with foul-smelling vaginal discharge. Also, we could find a focal area of vaginal inflammation in all patients. Six patients with RVF due to CD were concomitantly treated with 5 mg/kg of infliximab given as an intravenous induction regimen at 0, 2, and 6 weeks, followed by a maintenance regimen of 10 mg/kg every 8 weeks thereafter for the treatment of fistulizing CD.

**Fig. 1:**
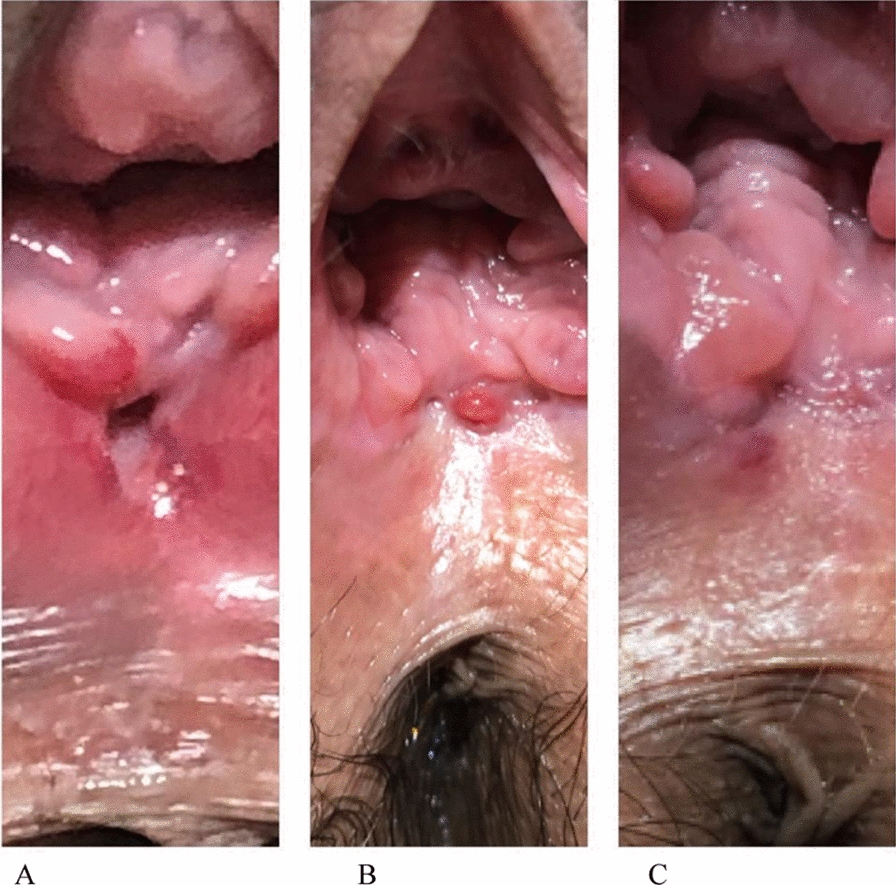
(Patient 9)—Photography shows fistulous orifice in the posterior vaginal wall before therapy (**A**). Photos **B** and **C** show clear reduction in the dimensions of the fistulous track after vaginal laser therapy

Overall, all patients completed five sessions of the vaginal laser therapy, and no adverse events were reported by any of the patients during follow-up. At week 20, on clinical examination, no persistent inflammatory signs were observed in the vagina in the fifteen patients (Fig. 1). Ten (66.7%) patients achieved clinical remission after the end of planned vaginal laser sessions, while 5 (33.3%) patients reported significant improvement in symptoms, although they still had occasional foul-smelling minimal vaginal discharge or passage of gas from the vagina. Moreover, five patients who did not have previous sexual activity returned to regular sexual activity. Seven patients who had previously sexual activity reported a clear improvement after vaginal laser treatment and three patients did not notice any change in sexual activity. Looking specifically at the subgroup of women who had sexual activity at baseline, we found a significant increase in FSQ scores between weeks 20 to 24 following vaginal laser therapy compared with baseline (71.9 ± 10.9 *versus* 40.9 ± 20.4, respectively; p = 0.006), indicating better sexual performance/satisfaction [14, 15].

Three out of four ostomized patients had their ostomy reversed. In particular, all six patients with RVF secondary to CD reported a marked improvement in symptoms and sexual function. Four were asymptomatic and two reported only passage of gas or elimination of foul-smelling from the vagina (Table 1). When patients were asked about their satisfaction with vaginal laser treatment at week 20 after the end of the fifth vaginal laser sessions, all fifteen patients reported being satisfied (n = 3; [20%]) or very satisfied (n = 12; [80%]) with the results achieved with this treatment.

A reduction in the size of the fistula or its resolution was observed in all patients, as described in Table 1.

**Fig. 2:**
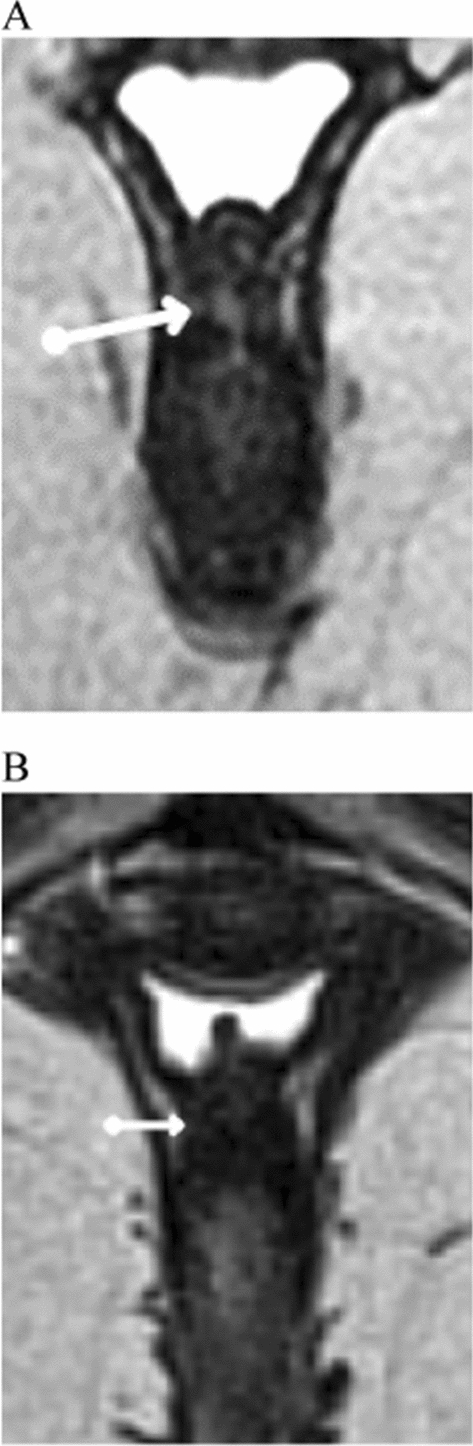
(Patient11)—Axial T2-weighted magnetic resonance image shows a fistulous track (white arrow) between the anal canal and the posterior vaginal wall (**A**). Magnetic resonance image after laser therapy no longer identifies the fistulous track with presence of low signal intensity tissue on T2-weighted images (white arrow), inferring fibrosis (**B**)

**Fig. 3:**
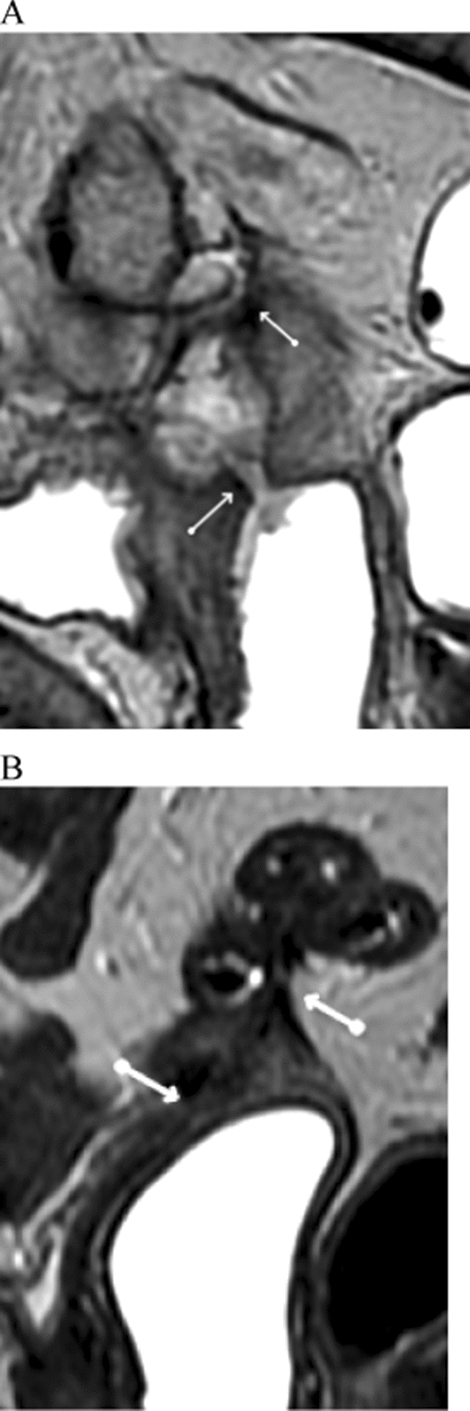
(Patient 12)—Sagittal T2-weighted magnetic resonance image shows fistulous track (white arrows) communicating the upper third of the vagina with abscesses in the pelvic cavity and intestinal loops (**A**). Magnetic resonance image after laser therapy no longer identifies the fistulous track with presence of low signal intensity tissue on T2-weighted images (white arrows), inferring fibrosis (**B**)

Seven patients (47%) achieved radiological remission between weeks 20 to 24 after ending the five sessions of vaginal laser, either with complete obliteration of the vaginal component of the fistula and presence of tissue proliferation or non-identification of the fistulous track, with the presence of tissue with low signal intensity on T2-weighted images, inferring fibrosis (Figs. 2 and 3). In addition, other eight patients (53%) had a reduction in the dimensions of the fistulous track.

## Discussion

Rectovaginal fistulas present a distressing problem for the patient and a challenge for the treating physician. Successful management must take into consideration the etiology of the fistula and the health of both the rectum and the patient [4]. For women with small fistulas and minimal symptoms, nonsurgical management is appropriate [7]. However, for the majority of patients with RVF, the symptoms are intolerable, and no standard treatment is accepted worldwide [16–20]^.^ Most published studies are small case series, which makes comparison of techniques and outcomes difficult [9].

Currently we have surgical approaches to anovaginal fistula or RVF repair, such as advancement flaps (endorectal and endovaginal), transperineal closure, Martius procedure, gracilis muscle transposition, rectal resections, transabdominal closure, mesh repair, plugs, endoscopic repairs (video-assisted anal fistula treatment -VAAFT, fistula laser closure—FiLaC, over-the-scope clip—OTSC—device) and closure with biomaterials [4, 7–9, 16–20]. The grade recommendation in treatment guideline approved by the ASCRS (The American Society of Colon & Rectal Surgeons) may be weak or strong [5] and we don’t have 100% of resolution.

In the search for treatment alternatives for RVF, accumulating evidence suggests that the fractional CO2 vaginal laser therapy could have a potential therapeutic role in this setting. Indeed, after the pilot study and a case series developed by Drumond et al. [12, 13] presenting potentially complementary, promising, and safe therapeutic strategy with fractional CO2 laser, new studies are being developed.

In present study, we found clinical and radiological remission rates of 66.7% and 47%, respectively between weeks 20 to 24 following fractional CO2 vaginal laser therapy. Additionaly, three patients had their ostomies successfully reversed and remained symptom-free during follow-up.

Interesting, despite the complexity of sexual function, we observe a significant increase in FSQ scores at the end of the treatment in most women and five patients who did not have previous sexual activity returned to regular sexual activity. In current study, eight out of 15 women studied maintained sexual activity during treatment. The medical care team chose not to recommend stopping sexual intercourse if patients so desired due to the long course of illness of most women included in the research. Furthermore, we believe that the advice to suspend sexual activity could worsen the suffering they experience, compromising interpersonal and family relationships. Furthermore, it is well known that postcoital non obstetric vaginal lacerations due to consensual sexual act are generally minute mucosal tears while acute fistula formation by penile penetration through the full thickness of the rectovaginal wall after consensual vaginal intercourse is very unusual [21]. Also, all patients reported being satisfied (20%) or very satisfied (80%) with the benefits obtained from the therapy. Sexual disfunction in females has a significant negative impact on a woman's health, self-esteem, relationships, quality of life, and work productivity [19–24]. Thus, therapeutic strategies that enable the return of sexual activity for the couple certainly contribute to improving the quality of life and other problems in woman’s health. Taken together, these findings strongly suggest that fractional CO2 vaginal laser therapy has the potential to induce clinical and radiological remission in a subgroup of patients with RVF, in addition to providing an important improvement in the sexual function of most women undergoing this treatment.

Patients with severe anorectal fistulizing CD who do not respond adequately to medical therapy, local surgical intervention or long-term seton drainage may consider fecal diversion with or without proctectomy to control anorectal sepsis and improve incontinence symptoms and overall quality of life [24]. Retrospective reviews evaluating diversion under these circumstances demonstrate that 64% to 81% of patients have an initial response to this approach [25]. In this study, 67% patients with CD were asymptomatic and 33% reported only passage of gas or elimination of foul-smelling from the vagina after vaginal laser therapy associated with infliximab continuous use. Thus, this combined therapeutic strategy of vaginal laser with anti-TNF therapy appears to be promising for the treatment of RVF in women with CD. Interestingly, the response to treatment was better in the group of patients with surgical fistulas than in those with RVF due to Crohn's disease. It is well recognized that RVF constitutes one of the most debilitating and refractory manifestations of perianal CD to treat, presenting high rates of recurrence and frequent need for multiple consecutive therapies. Indeed, the decreased vascularization and the thin rectovaginal septum do not provide ideal conditions to promote healing, probably justifying the greater difficulty in obtaining complete RVF healing in patients with Crohn's disease [26]. Of note, in our series no adverse event of vaginal laser therapy was noticed. It can be performed on an outpatient basis and without anesthesia.

This research provides new therapeutic perspectives for the application of this technique in RVF repair. However, long-term prospective studies involving a broader population of women with RVF are needed to confirm the preliminary findings of this innovative therapy. In addition, future studies it would be important to test new CO2 laser treatment as an adjuvant therapy in patients with RVF since this tool demonstrated potential beneficial effects on the vaginal mucosa with regard to other pathologies [27].

## Conclusions

Treatment with fractional CO2 vaginal laser has proven capable of inducing clinical and radiological remission in a significant proportion of patients with RVF. We found a reduction of vaginal inflammatory process in all patients as well as improvement in sexual function as evaluated by the FSQ. No adverse effects were identified in medium term follow up. Further prospective studies with appropriate simple size are needed to establish this method as a complementary or first-line therapeutic strategy for patients with RVF.

### Limitations

The main limitations of this research refer to the limited possibility of generalizing the validity of the study due to the sample size due to the rarity of the condition.

## Data Availability

The datasets used and/or analyzed during the current study are available from the corresponding author on reasonable request.
